# WNT5A–ROR2 is induced by inflammatory mediators and is involved in the migration of human ovarian cancer cell line SKOV-3

**DOI:** 10.1186/s11658-016-0003-3

**Published:** 2016-07-28

**Authors:** Somayeh Arabzadeh, Ghamartaj Hossein, Zahra Salehi-Dulabi, Amir Hassan Zarnani

**Affiliations:** 1 Department of Animal Physiology, Developmental Biology Laboratory, School of Biology, College of Science, University of Tehran, Tehran, Iran; 2 Nanobiotechnology Research Center, Avicenna Research Institute, ACECR, Tehran, Iran; 3 Immunology Research Center, Iran University of Medical Sciences, Tehran, Iran

**Keywords:** Ovarian cancer, Inflammation, Wnt5A, ROR2, NF-kB/STAT3 signaling pathways, Migration

## Abstract

**Background:**

Wnt5A, which is a member of the non-transforming Wnt protein family, is implicated in inflammatory processes. It is also highly expressed by ovarian cancer cells. ROR2, which is a member of the Ror-family of receptor tyrosine kinases, acts as a receptor or co-receptor for Wnt5A. The Wnt5A–ROR2 signaling pathway plays essential roles in the migration and invasion of several types of tumor cell and influences their cell polarity. We investigated the modulation of Wnt5A–ROR2 by inflammatory mediators and its involvement in the migration of the human ovarian cancer cell line SKOV-3.

**Methods:**

SKOV-3 cells were treated with LPS (lipopolysaccharide), LTA (lipoteichoic acid) and recombinant human IL-6 alone or in combination with STAT3 inhibitor (S1155S31-201) or NF-kB inhibitor (BAY11-7082) for 4, 8, 12, 24 and 48 h. The Wnt5A and ROR2 expression levels were determined at the gene and protein levels. Cells were transfected with specific siRNA against Wnt5A in the absence or presence of human anti-ROR2 antibody and cell migration was assessed using transwells.

**Results:**

There was a strong downregulation of Wnt5A expression in the presence of STAT3 or NF-kB inhibitors. Cell stimulation with LTA or IL-6 for 8 h led to significantly increased levels of Wnt5A (5- and 3-fold higher, respectively). LPS, LTA or IL-6 treatment significantly increased ROR2 expression (2-fold after 48 h). LPS- or LTA-induced Wnt5A or ROR2 expression was abrogated in the presence of STAT3 inhibitor (*p* < 0.001). IL-6-induced Wnt5A expression was abrogated by both STAT3 and NF-kB inhibitors (*p* < 0.001). Although not significant, IL-6-induced ROR2 expression showed a modest decrease when STAT3 inhibitor was used. Moreover, cell migration was decreased by 80 % in siRNA Wnt5A-transfected cells in the presence of anti-human ROR2 antibody (*p* < 0.001).

**Conclusions:**

This study revealed for the first time that inflammatory mediators modulate Wnt5A and ROR2 through NF-kB and STAT3 transcription factors and this may play a role in ovarian cancer cell migration. The results described here provide new insight into the role of the Wnt5A–ROR2 complex in ovarian cancer progression in relation to inflammation.

## Background

Ovarian cancer is the seventh most common cancer and the eighth most common cause of cancer deaths among women worldwide [1]. More than 85 % of ovarian cancers are of epithelial origin and the majority of deaths are attributed to the serous carcinoma sub-type [2]. Approximately 80 % of patients with primary disease respond to surgery and chemotherapy, but 60–80 % of these patients will present with recurrent disease 6 months to 2 years post-treatment [2].

The immune system normally causes an inflammatory reaction to kill the abnormal tumor cells. There is increasing evidence that inflammation can also enhance the growth of tumor cells that have “learned” to use the inflammatory process in a way that benefits their development. It has been demonstrated that in addition to associations between inflammatory conditions and particular human tumors, there is a direct link between chronic inflammation and tumorigenesis, particularly through activation of the NF-kB and STAT3 signaling pathways [3].

Wingless proteins, termed Wnt, are a family of cysteine-rich glycoproteins that regulate embryonic development, cell proliferation, differentiation, migration and death [4]. Wnt5A plays essential roles in developmental and physiological processes, including inflammation, but it also plays a role in cancer [5–8]. Its role in inflammation was demonstrated when it was shown to be expressed by monocytes and endothelial cells and modulated by microbial stimulation [7, 8]. Wnt5A primarily activates the β-catenin-independent pathway of Wnt signaling, which is known to be a non-canonical pathway where ROR2, a member of the Ror-family of receptor tyrosine kinases, may act as a receptor or co-receptor for Wnt5A [5]. Wnt5A–ROR2 signaling involves Disheveled (Dvl), JNK⁄AP-1, the Src family of non-receptor protein tyrosine kinases and Ca^2+^. It plays essential roles in the migration and invasion and influences the cell polarity of several types of tumor cell [5]. Previous studies on the interactions between ROR2 and Wnt signaling have indicated that ROR2 has multiple functions depending on the cellular context [9]. In recent years, the interaction between ROR2 and Wnt5A in cancer has received great attention. It has been shown that sustained or increased expression of Wnt5A and/or ROR2 affects the invasive properties of several types of tumor cell, including melanoma, osteosarcoma, renal cell carcinoma, prostate carcinoma, gastric cancer and pancreatic cancer [10–15].

In this study, we aimed to understand whether inflammatory mediators such as lipopolysaccharide (LPS), lipoteichoic acid (LTA) or recombinant human IL-6 (rhIL-6) can modulate Wnt5A and ROR2 expression in the human ovarian cancer cell line SKOV-3. In addition, we assessed the role of STAT3 and NF-kB transcription factors in the regulation of Wnt5A and ROR2 expression and determined whether the Wnt5A–ROR2 complex could influence SKOV-3 cell migration.

## Methods

### Cells and treatment

Cells of the human ovarian carcinoma cell line SKOV-3 were donated by Dr. A.H. Zarnani (Avicenna Research Center, Tehran). The cells were grown in RPMI-1640 medium (Gibco BRL) supplemented with 10 % fetal bovine serum (FBS) and penicillin/streptomycin antibiotics obtained from Life Technologies GmbH. Growth conditions were 37 °C in a 5 % CO_2_ atmosphere with 90–95 % humidity.

The cells were treated with the following inflammatory mediators: 1 μg/ml LPS; 30 μg/ml LTA (Sigma-Aldrich); or 100 ng/ml rhIL-6 (Peprotech). Treatment ran for 4, 8, 12, 24 and 48 h before the assessment of Wnt5A and ROR2 expression levels.

In order to assess the role of NF-kB and STAT3 transcription factors on Wnt5A and ROR2 expression, the cells were treated with 5 and 10 μM NF-kB inhibitor (BAY11-7082; Reagentsdirect) or 25 and 50 μM STAT3 inhibitor (S31-201; Selleck Chemical). The involvement of NF-kB or STAT3 transcription factors on inflammation-induced Wnt5A or ROR2 expression was assessed using 10 μM BAY11-7082 or 25 μM S31-201, which were added 1 h before the addition of the inflammatory mediators.

### Cell survival assay

Cell survival was determined using the MTT assay. SKOV-3 cells were seeded at 8000 cells/well in 96-well plates in medium plus 10 % FBS for 24 h. Cells were treated with or without BAY11-7082 (5, 10 μM) or S31-201 (25, 50 μM) for 48 h and cell survival was assessed as previously described [16].

### Quantitative RT-PCR

Total RNA was extracted using RNX-Plus reagent (Cinnagen). Then, 1 μg of the RNA was converted to cDNA using RevertAid Reverse Transcriptase (Fermentas). Real-time PCR was conducted using 1x SYBR Premix Ex Taq II (Tli RNaseH Plus; Takara) on an iCyler IQ5 (Bio-Rad) using a continuous fluorescence-detecting thermal cycler (Rotor-Gene Q; Qiagen).

The sequence of the primers used was:

- Wnt5A – forward: 5′-GCCATGAAGAAGTCCATTG-3′; reverse: 5′-AGCGACCACCAAGAATTG-3′;
- 18sRNA – forward: 5′-GTAACCCGTTGAACCCCATT-3′; reverse: 5′-CCATCCAATCGGTAGTAGCG-3′.

Quantification was performed via the standard curve method using REST-RG software version 3, and the data were normalized relative to the 18 s RNA housekeeping gene. Experiments were performed in triplicate.

### Western blot

The concentrations and sources of the antibodies were as follows: mouse monoclonal anti-human Wnt5A antibody (1:1500, Abcam), rabbit polyclonal anti-ROR2 (1:1500, Origene) and rabbit polyclonal anti-GAPDH (1:1000, Abcam). Western blot was performed as previously described [16]. Bands were quantified with densitometric analysis, using AlphaEaseFC software. Results were expressed relative to the untreated cells (control, set as 1.0).

### SiRNA transfection

The specific and negative (scramble) controls were Wnt5A knocked down using small interfering RNA (siRNA; ON-TARGET plus SMART pool human Wnt5A, Fisher Scientific AG) and non-target siRNAs (ON-TARGET plus SMART pool human NonTarget siRNA, Fisher Scientific AG). Cells treated with only the transfection reagent lipofectamine 2000 (Invitrogen) were used as an additional control. To inhibit endosomal acidification, the cells were pre-treated with 100 μM chloroquine (Invitrogen) for 30 min before transfection. Cells were transfected at 30–50 % confluence using lipofectamine 2000 to give a final siRNA concentration of 25 nM for 8 h without FBS and antibiotics. Then, the media were replaced with media containing 10 % FBS plus antibiotics for 48 h.

### Migration assay

Control, siRNA scramble and siRNA Wnt5A-transfected cells were trypsinized after 48 h. The cells were seeded on the upper chamber of an 8 μm pore size transwell insert (Costar) at 2.5 × 10^4^ cells/insert in RPMI-1640. The lower chamber contained RPMI-1640 culture solution (600 μl) plus 10 % FBS. Anti-human ROR2 antibody (Origene) or rhIL-6 (100 ng/ml) were added to the upper chamber 30 min after seeding and incubated for 5 h at 37 °C. Non-migratory cells on the upper surface of the membrane were removed and cells were fixed with 4 % paraformaldehyde in PBS and stained in 0.5 % crystal violet. The membranes were mounted on a microscope slide. Migrated cells were counted in ten random fields with a light microscope (Zeiss). Photographs were taken with an AxioCam digital camera (Zeiss). The percentage of migrated cells was calculated. The migration index was expressed relative to control cells (set as 100 %). All the experiments were carried out three times and results were expressed as the means ± SD.

### Statistical analysis

Normality of nominal variables was analyzed using the Kolmogorov Smirnov test. Data were analyzed with the statistical software package SPSS 19.0 (SPSS Inc.). We used an unpaired 2-tailed Student’s *t* test or, for comparison of data among groups, 1-way ANOVA test. *p* < 0.05 was considered statistically significant.

## Results

### Wnt5A and ROR2 expression are induced by LPS, LTA and IL-6

**Fig. 1:**
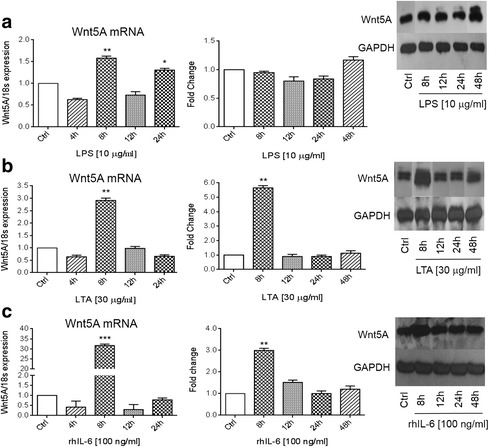
Wnt5A expression induced by LPS, LTA and IL-6. SKOV-3 cells were treated with 1 μg/ml LPS (**a**); 30 μg/ml LTA (**b**) or 100 ng/ml IL-6 (**c**) for the indicated times. The left panels show normalized values (means ± SD) from three independent quantitative PCR analyses for Wnt5A expression. Data were normalized related to 18 s RNA as the internal control. The right panels show normalized values (means ± SD) from three independent western blots for Wnt5A. The western blots represent one of three independent experiments. GAPDH levels were used as the internal control. *p ≤ 0.05; **p ≤ 0.01; ***p ≤ 0.001 as compared with the control (Ctrl)

**Fig. 2:**
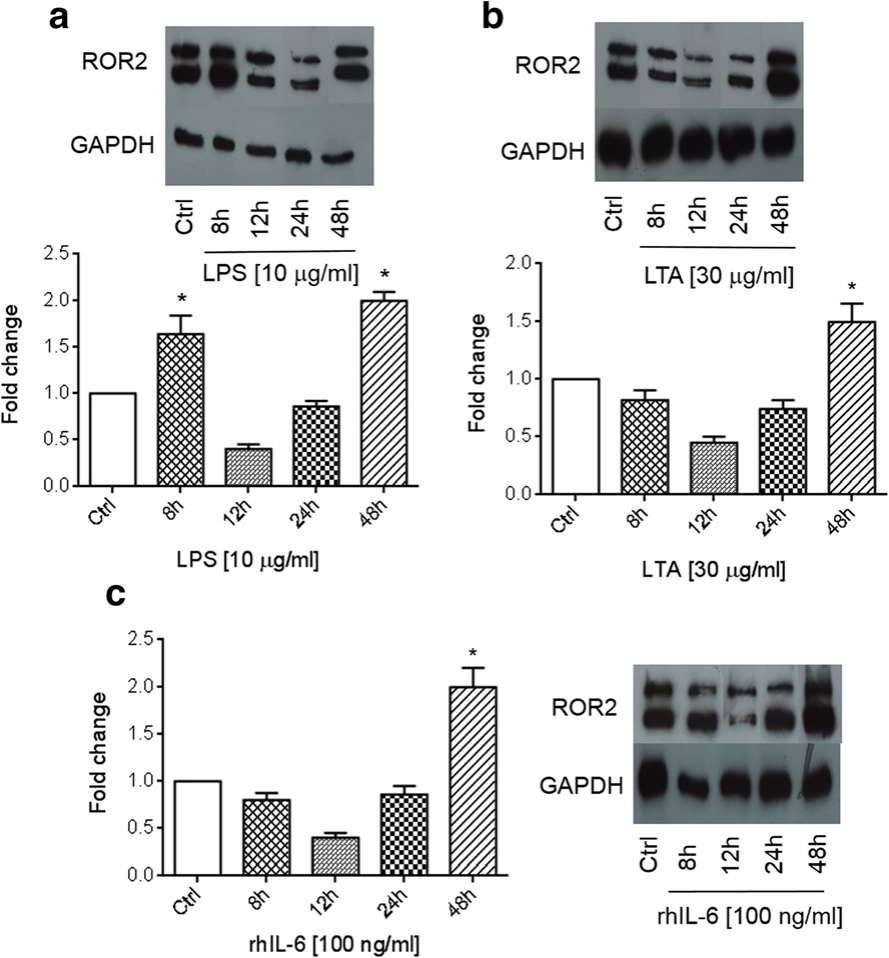
ROR2 expression induced by LPS, LTA and IL-6. Western blot analysis of ROR2 expression in SKOV-3 cells treated with 1 μg/ml LPS (**a**), 30 μg/ml LTA (**b**) and 100 ng/ml IL-6 (**c**) for the indicated times. Normalized values (means ± SD) from three independent western blots for ROR2. The western blots represent one of three independent experiments. GAPDH levels were used as the internal control. *p ≤ 0.05 as compared with the control (Ctrl)

To study the modulation of Wnt5A–ROR2 signaling by inflammatory mediators, cells were stimulated with LPS, LTA and IL-6 for 4, 8, 12, 24 and 48 h. Real-time PCR analysis showed a significant 1.5-, 3- and 30-fold increase in the Wnt5A mRNA levels after 8 h stimulation with LPS, LTA and IL-6, respectively (Fig. 1, left panel). These results were confirmed at the protein level for LTA and IL-6 (Fig. 1, right panel). There was no significant increase in the Wnt5A protein level after LPS stimulation (Fig. 1a, right panel). Western blot analysis showed significant increases in the ROR2 protein level: 2-fold in the presence of LPS or IL-6 and 1.5-fold with LTA after 48 h (Fig. 2).

### Wnt5A–ROR2 expression is mediated by NF-kB and STAT3 signaling pathways

**Fig. 3:**
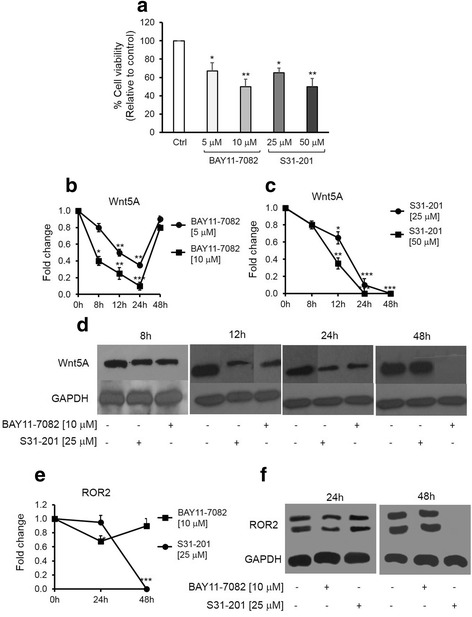
NF-kB and STAT3 inhibitors alter SKOV-3 cell viability and affect Wnt5A and ROR2 expression. **a** – MTT assay of cell viability in the presence of BAY11-7082 as an NF-kB inhibitor (5, 10 μM) or S31-201 as a STAT3 inhibitor (25, 50 μM). **b** – Normalized values (means ± SD) from three independent western blots for Wnt5A in the presence of BAY11-7082 for the indicated times. **c** – Normalized values (means ± SD) from three independent western blots for Wnt5A in the presence of S31-201 for the indicated times. D – Western blot analysis of Wnt5A in cells treated with BAY11-7082 (5, 10 μM) or S31-201 (25, 50 μM) for the indicated times. The western blots represent one of three independent experiments. E – Normalized values (means ± SD) from three independent western blots for ROR2 in the presence of BAY11-7082 or S31-201 for 24 and 48 h. F- Western blot analysis of ROR2 in cells treated with BAY11-7082 (5 μM) or S31-201 (25 μM) for 24 and 48 h. The western blots represent one of three independent experiments. GAPDH levels were used as the internal control. *p ≤ 0.05; **p ≤ 0.01; ***p ≤ 0.001 as compared with the control (Ctrl)

NF-kB and STAT3 have been implicated in the regulation of Wnt5A transcription [17, 18]. However, the role of these transcription factors in the regulation of Wnt5A in human ovarian cancer remains unknown. We assessed the cytotoxic effect of BAY11-7082 as an NF-kB inhibitor (5 and 10 μM) or S31-201 as a STAT3 inhibitor (25 and 50 μM) on SKOV-3 cells. There were 37 % (*p* < 0.05) and 50 % (*p* < 0.01) decreases in cell viability in the presence of 5 or 10 μM BAY11-7082, respectively. Similarly, there were 40 % (*p* < 0.05) and 50 % (*p* < 0.01) decreases in cell viability in the presence of 25 or 50 μM S31-201, respectively (Fig. 3a). Western blot analysis showed that Wnt5A expression decreased in the period from 8 to 24 h in the presence of BAY11-7082 (Fig. 3b and d). In the presence of S31-201, Wnt5A expression decreased in the period from 12 to 48 h (Fig. 3c and d). Based on these data, the effect of inhibitors on ROR2 expression level was assessed at 24 and 48 h. The results showed that BAY11-7082 led to a 30 % decrease in ROR2 at 24 h (*p* < 0.05), while S31-201 abrogated ROR2 expression (*p* < 0.001) at 48 h (Fig. 3e and f).

### NF-kB and STAT3 signaling pathways are implicated in inflammation-induced Wnt5A and ROR2 expression

**Fig. 4:**
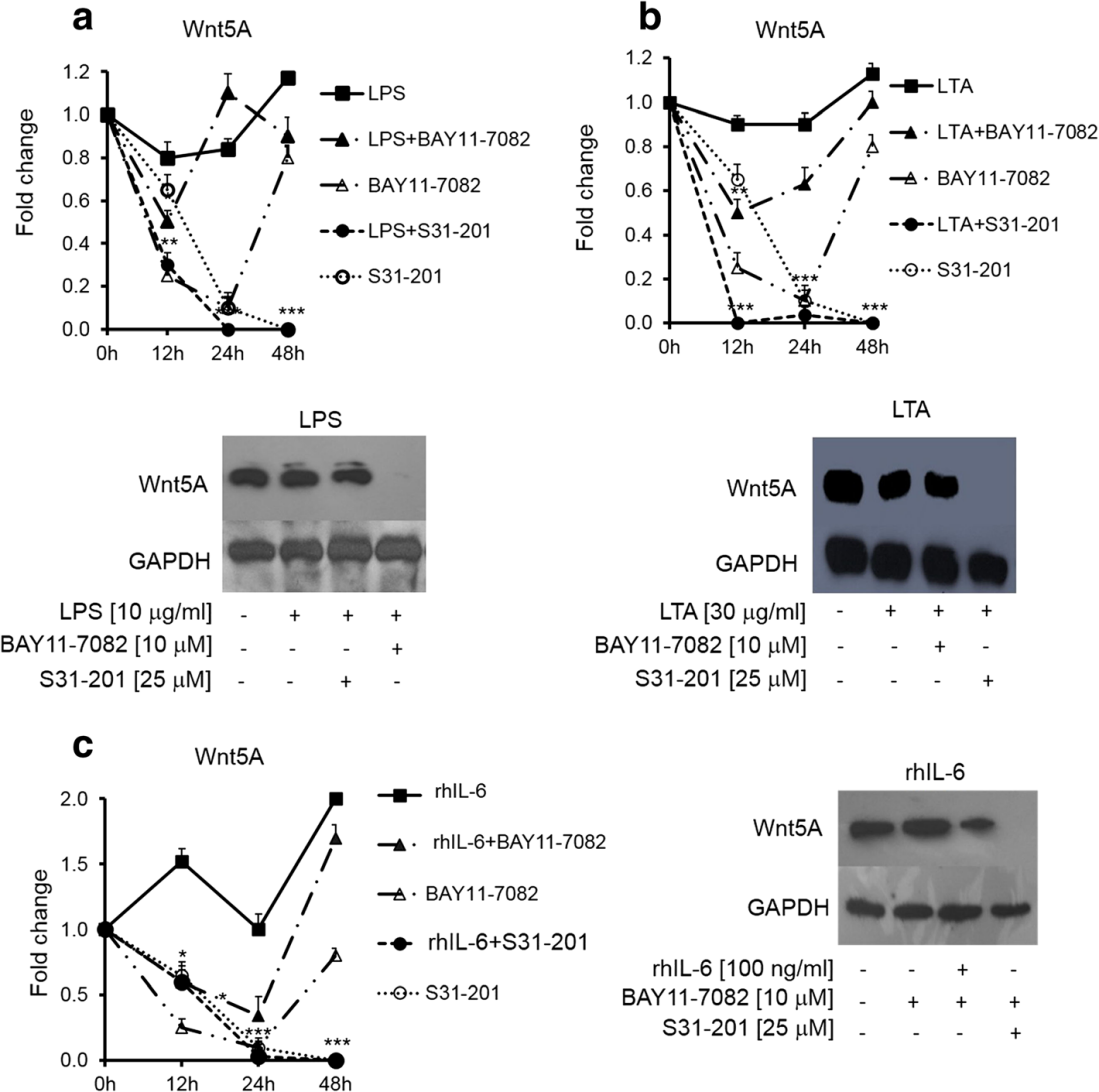
Involvement of NF-kB and STAT3 signaling pathways in inflammation-induced Wnt5A expression. SKOV-3 cells treated with inflammatory mediators alone or in combination with BAY11-7082 as an NF-kB inhibitor or S31-201 as a STAT3 inhibitor for 24 h. LPS (**a**) and LTA (**b**). The upper panels show normalized values (means ± SD) from three independent western blots for Wnt5A expression for the indicated times. The lower panels represent one of three independent western blot analysis of Wnt5A. **c** – rhIL-6, with the left panel showing normalized values (means ± SD) from three independent western blots for Wnt5A expression for the indicated times and the right panel representing one of three independent western blot analyses of Wnt5A. GAPDH levels were used as the internal control. *p ≤ 0.05; **p ≤ 0.01; ***p ≤ 0.001 as compared with the control (Ctrl)

**Fig. 5:**
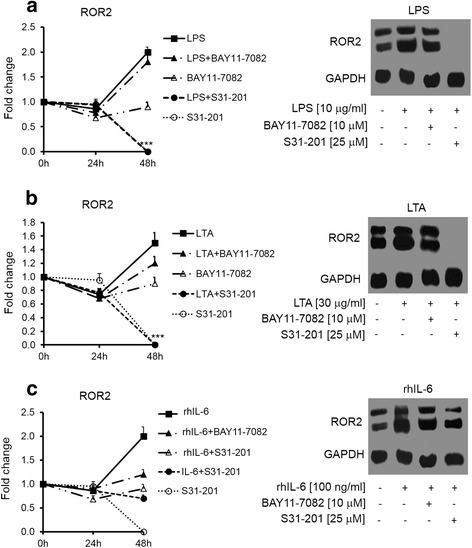
Involvement of NF-kB and STAT3 signaling pathways in inflammation-induced ROR2 expression. The left panels show normalized values (means ± SD) from three independent western blots for ROR2 expression for the indicated times and the right panels represent one of three independent western blot analyses of ROR2 in SKOV-3 cells treated with LPS (**a**); LTA (**b**) and rhIL-6 (**c**) alone or in combination with BAY11-7082 or S31-201 for 48 h. GAPDH levels were used as the internal control. ***p ≤ 0.001 as compared with the control (Ctrl)

NF-kB and STAT3 are known to mediate the expression of many genes that influence growth and inflammation and are often upregulated in cancer [3]. Therefore, we assessed the effect of inflammatory mediators in the presence or absence of above-mentioned NF-kB and STAT3 inhibitors. LPS- and LTA-induced Wnt5A expression was abrogated with the STAT3 inhibitor (Fig. 4a and b, upper and lower panels) and IL-6-induced Wnt5A expression was modulated by both STAT3 and NF-kB signaling pathways (Fig. 4c, right and left panels). Similarly, LPS- and LTA-induced ROR2 expression was abrogated in the presence of the STAT3 inhibitor (Fig. 5a and b, right and left panels). IL-6-induced ROR2 expression remained unchanged in the presence of the inhibitors (Fig. 5c).

### The Wnt5A–ROR2 complex is involved in migration of the SKOV-3 cell line

**Fig. 6:**
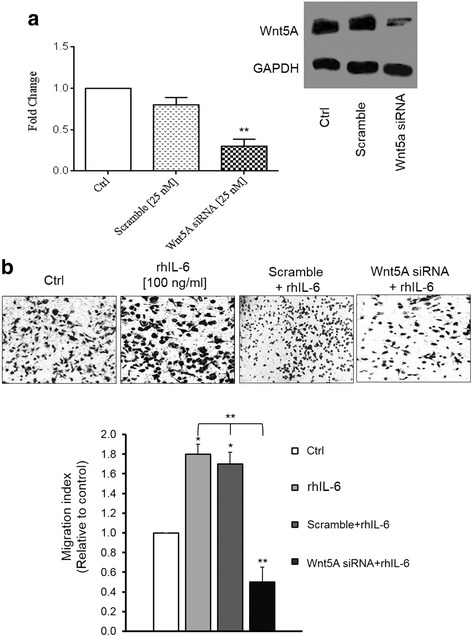
Wnt5A is involved in rhIL-6-induced migration in SKOV-3 cells. **a** – Western blot quantification showed a 70 % reduction in Wnt5A expression in siRNA Wnt5A-transfected cells compared with the controls. The western blots represent one of three independent experiments. GAPDH levels were used as the internal control. **b** – In vitro cell migration assay of non-transfected or siRNA Wnt5A-transfected cells in the absence or presence of rhIL-6. The upper panel showed photos of transwells in the indicated conditions and the lower panel showed quantification of migrated cells in ten random fields. The percentage of migrated cells was expressed as: *(number of migrated cells/number of seeded cells × 100)*. The migration index was expressed relative to control cells (set as 100 %). All of the experiments were carried out three times and the results are expressed as means ± SD. Magnification: 100x. *p ≤ 0.05; **p ≤ 0.0 as compared with the control (Ctrl)

Our previous study [16] showed that SKOV-3 cell migration increased in Wnt5A-overexpressing SKOV-3 cells. Having shown that rhIL-6 led to increased levels of Wnt5A expression (Fig. 1), we assessed whether Wnt5A may be implicated in IL-6-induced cell migration. Wnt5A expression level was modulated using siRNA Wnt5A transfected cells that showed 70 % decreased Wnt5A levels (Fig. 6a). Moreover, Wnt5A knockdown cells were used for the migration assay in the absence or presence of rhIL-6. There was 1.8- fold increase in cell migration with rhIL-6 (*P* < 0.05), whereas Wnt5A knockdown cells that were treated with rhIL-6 showed a 50 % decrease in cell migration relative to the control (*p* < 0.001; Fig. 6b, upper and lower panels).

**Fig. 7:**
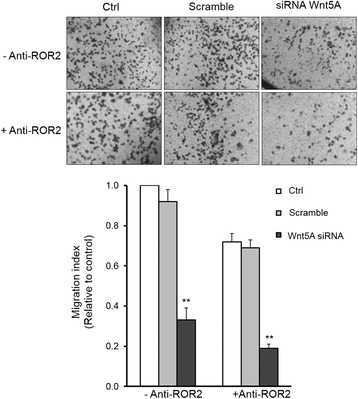
Wnt5A–ROR2 is implicated in SKOV-3 cell migration. **a** – In vitro cell migration assay of non-transfected or siRNA Wnt5A-transfected cells in the absence or presence of anti-ROR2 antibody. The upper part or photos shows photos of transwells with the indicated conditions. The graph shows quantification of counted migrated cells in ten random fields. All of the experiments were carried out three times and the results are expressed as means ± SD. Magnification: 100x. **p ≤ 0.01 as compared with the control (Ctrl)

Previous studies showed that Wnt5A requires ROR2 expression to mediate the migration of cells during mammalian palate development [19] and that increased expression of Wnt5A led to increased melanoma cell motility [10]. Therefore, it was tempting to speculate that Wnt5A–ROR2 may be involved in the migration of ovarian cancer cells. The migration assay revealed that knockdown of Wnt5A expression influenced cell migration: there was a 67 % decrease in cell migration in siRNA Wnt5A-transfected cells compared to the controls (*p* < 0.01). Moreover, adding anti-ROR2 antibody in the culture media led to a 29 % reduction in cell migration in the controls and an 80 % decrease in cell migration in siRNA Wnt5A-transfected cells (Fig. 7, upper and lower panels). These results showed that Wnt5A mediates SKOV-3 cell migration through ROR2 signaling.

## Discussion

It has been demonstrated that Wnt5A is involved in inflammatory processes but its role in ovarian cancer in relation to inflammation remains unknown. Several studies showed the role of Wnt5A in inflammation-related diseases [20, 21] and highlighted the relation between the Wnt5A signaling pathway and cytokine production in cells of monocytic lineage [7, 22], neutrophils [23] and endothelial cells [24]. In the ovary, Wnt5A acts as a pro-inflammatory factor in the ovarian granulosa cells of women with polycystic ovarian syndrome [25].

In support of a role for Wnt5A in inflammation, previous studies showed that NF-kB has been implicated in the regulation of Wnt5A transcription [26, 27]. A STAT3-binding site was not identified within the human Wnt5A exonic regions but tandem STAT3-binding sites with 11-bp spacing were identified within the conserved region in intron 4 [18]. Wnt5A activity in pathological inflammation has been linked to Toll-like receptors (TLRs), a family of transmembrane signaling proteins that trigger the innate immune response [7].

In addition, it has been reported that healthy ovary tissue, human epithelial tumors and numerous ovarian cancer cell lines, including SKOV-3 cells, express TLR2, -3, -4 and -5 and IL-6 receptor [28, 29]. It was suggested that activation of these receptors initiates NF-kB and/or STAT3 activation, which may constitute a mechanism by which the cancerous epithelial cells can manipulate inflammatory pathways to encourage tumor growth [28–30]. It is hypothesized that constitutive NF-kB signaling defines a subset of ovarian cancer, susceptible to therapeutic targeting of this pathway [31, 32].

Our previous study and other studies demonstrated a promoting role of Wnt5A in epithelial ovarian cancer [16, 33–36]. However, one study reported that Wnt5A supresses epithelial ovarian tumor formation by promoting surface epithelial cell senecsence [37]. To date, this has not been confirmed further. ROR2 may be a receptor or co-receptor of Wnt5A in a context-dependent manner [6]. It showed high expression in osteosarcoma [11] and renal cell carcinoma [12] and its high expression in neuroblastoma was correlated with poorer survival [38]. To the best of our knowledge, there are no data about the modulation of Wnt5A–ROR2 by inflammatory mediators in ovarian cancer cells. Here, we showed upregulation of Wnt5A expressionby LPS, LTA and to a larger exent by IL-6 in SKOV-3 cells.

In ovarian cancer, the increased activity of NF-kB has been reported to confer resistance to chemotherapeutic agent-induced apoptosis [39, 40]. Moreover, it has been demonstrated that blockage of NF-kB activity in SKOV-3, ip1 and HEYA8 ovarian cancer cells can reduce tumor growth in xenograft mice [41]. In addition, blockage of the JAK/STAT3 pathway inhibited ovarian cancer cell growth [42]. In agreement with these findings, we found here that NF-kB and STAT3 inhibitors decrease SKOV-3 cell viability.

It was intersting to find that both NF-kB and STAT3 inhibitors decrease Wnt5A expression in a time-dependent manner. However, our data showed that blockage of STAT3 activation has the strongest effect on the modulation of Wnt5A and ROR2 expression compared to treatment of cells with NF-kB inhibitor.

This is the first report showing the expression of ROR2 in SKOV-3 cells and its modulation by inflammatory mediators. Similar to Wnt5A, ROR2 expression was increased by LPS, LTA and IL-6. Its expression was moderately decreased by the NF-kB inhibitor but strongly blocked by the presence of STAT3 inhibitor.

Although Wnt5A–ROR2 expression was regulated by both NF-kB and STAT3, only LPS- and LTA-induced Wnt5A–ROR2 increases were completely abrogated by STAT3 inhibitor. The IL-6-induced Wnt5A increase was abrogated in the presence of STAT3 inhibitor, but IL-6-induced ROR2 was only moderately decreased with the STAT3 inhibitor. We can not exclude the possibility that IL-6 may trigger other signaling pathways that further influence ROR2 expression. It should be noted that to date there was no report on the existence of an NF-kB- or STAT3-binding site in the ROR2 promoter.

Interestingly, we observed that Wnt5A levels regulate ROR2 expression level. This means that following downregulation of Wnt5A expression, there was strong decrease in ROR2 expression levels (unpublished data). This is also supported by our data showing that the ROR2 response to inflammatory mediators takes more time than the Wnt5A response. This may suggest that the effect of NF-kB and STAT3 on ROR2 modulation could be mediated indirectly via Wnt5A levels. A downstream Wnt5A target that affects ROR2 expression levels remains unknown.

Two mechanisms are under consideration for the strongest effect of the STAT3 inhibitor on the Wnt5A–ROR2 expression level. As SKOV-3 cells have NF-kB constitutive activation [43], one possibility is that activated STAT3 in these cells can ensure constitutive NF-kB activation [44]. Another mechanism may be that once in the nucleus, the NF-kB–STAT3 complex can bind to unique Wnt5A or ROR2 DNA target sequences to which neither factor can bind on its own. These hypotheses warrants furher experiments.

It is well known that inflammation mediates cancer cell migration [3]. In our previous study, we showed that Wnt5A induced SKOV-3 cell migration [16]. It has been reported that IL-6 promotes EOC cell proliferation, migration and invasion [45, 46]. Here, we showed that Wnt5A is involved in IL-6 increases in cell migration.

## Conclusions

Our data showed for the first time that Wnt5A–ROR2 is modulated by inflammatory mediators and inflammatory pathways, and this prompted us to assess the role of Wnt5A–ROR2 in ovarian cancer migration. This is the first report showing that ROR2 signaling is implicated in Wnt5A-induced cell migration, which corroborates the finding of Nishita et al. [47] in other cancer cells. The findings of this study may provide new insight into the role of Wnt5A–ROR2 in ovarian cancer progression and help in the development of further targeted drugs for ovarian cancer treatment.

## Abbreviations

FBS, Fetal Bovine Serum; IL-6, Interleukin-6; LPS, Lipopolysaccharide; LTA, Lipoteichoic Acid; NF-kB, Nuclear Factor KappaB; ROR2, Receptor tyrosine kinase-like Orphan Receptor 2; STAT3, Signal Transducer and Activator of Transcription 3; Wnt5A, Wingless-type MMTV integration site family member 5A
